# Therapeutic Efficacy of Great Plantain (*Plantago major* L.) in the Treatment of Second-Degree Burn Wounds: A Case-Control Study

**DOI:** 10.1155/2022/4923277

**Published:** 2022-08-01

**Authors:** Abdolkhalegh Keshavarzi, Hashem Montaseri, Rahimeh Akrami, Hossein Moradi Sarvestani, Fateme Khosravi, Sara Foolad, Mitra Zardosht, Saeid Zareie, Mohammad Jamal Saharkhiz, Reza Shahriarirad

**Affiliations:** ^1^Burn and Wound Healing Research Center, Shiraz University of Medical Sciences, Shiraz, Iran; ^2^Department of Quality Control, Faculty of Pharmacy, Shiraz University of Medical Sciences, Shiraz, Iran; ^3^MPH Department, School of Medicine, Shiraz University of Medical Sciences, Shiraz, Iran; ^4^Student Research Committee, Shiraz University of Medical Sciences, Shiraz, Iran; ^5^College of Agricultural Sciences, Shiraz Branch, Islamic Azad University, Shiraz, Iran; ^6^Bachelor of Nursing, Nurse of Intensive Care Unit (ICU) of Amir Al-Momenin Burn Injury Hospital, Shiraz University of Medical Sciences, Shiraz, Iran; ^7^Department of Horticultural Sciences, College of Agriculture, Shiraz University, Shiraz, Iran; ^8^Thoracic and Vascular Surgery Research Center, Shiraz University of Medical Sciences, Shiraz, Iran

## Abstract

**Background:**

Great plantain (*Plantago major* L. or *P.major*) is a medicinal plant that is available all around the world. The whole plant has several bioactive compounds including terpenoids, flavonoids, phenolic compounds, alkaloids, fatty acids, iridoid glycosides, polysaccharides, and vitamins. Scientific studies have recognized several medical benefits like wound healing, anti-inflammatory, antimicrobial, antiulcerative, and antioxidative agents. The wound-healing capacity of this plant has been investigated under *in vivo* and ex vivo conditions. In the current study, we aim to compare the therapeutic effect of the *P.major* extract with 1% sulfadiazine on the healing of second-degree burn wounds.

**Method:**

Second-degree burn victims were included in our study. The investigation and control group, respectively, received *P. major* ointment 10% and silver sulfadiazine ointment 1%. The bacterial culture from the wound site was taken on days 3, 7, 10, 13, and last day of hospitalization. Patients' subjective complaints were obtained through the visual analog scale (VAS). All patients were treated and evaluated in the hospital.

**Result:**

Among the 15 patients, 11 were male, and the mean age was 33.3 years. The average complete healing duration was 11.73 *vs*. 13 days in the *P. major* and control group, respectively (*P*=0.166). On the third day, infection control was similar between the two groups, and on the seventh day, all bacterial cultures were negative. Although there was a significant reduction in pain scores during the recovery time, no significant differences in pain reduction were noted between the two groups (*P*=0.849).

**Conclusion:**

We showed that *P.major* ointment is a safe and suitable herbal compound in the treatment of second-degree burn wounds that not only has wound-healing properties but also is an analgesic and antimicrobial compound.

## 1. Introduction

Throughout the world, burn injuries are one of the most catastrophic and common injuries of all, causing thousands of deaths annually in addition to long-lasting disabilities and poor quality of life among burn victims [1]. These injuries more frequently occur in developing and low-income countries rather than in more developed ones and put a heavy burden through direct and indirect healthcare costs, several surgeries, hospitalization, and rehabilitation [2–6]. Partial-thickness burns are one of the most frequent burn injuries that require daily wound washing and dressing with topical antimicrobial compounds. In superficial and partial-thickness burn therapy, silver sulfadiazine is introduced as the main topical therapy due to its antimicrobial properties. Its possible side effects like black spotting, sliver hypersensitivity, incomplete epithelialization, neutropenia, thrombocytopenia, and limitation of use under specific conditions like G6PD deficiency have put other topical compounds in perspective [5].

Herbal medicine and alternative therapies have always obtained a high interest in the management of diseases and conditions [7–9]. In this regard, *Plantago major* is a perennial plant that belongs to the Plantaginaceae family. This plant is native to most of Europe and northern and central Asia [10, 11]. In traditional medicine, this plant has been extensively used in a number of diseases related to ophthalmology, reproduction, pulmonology, gastrointestinal system, otorhinolaryngology, and dermatology [12]. In recent years, researchers have found several bioactive compounds and mechanisms that are responsible for the wide range of medical benefits of this plant. Flavonoid compounds that are isolated from the whole plant contain antidiabetic, antidiarrheal, anti-inflammatory, and wound-healing capacity [11]. Polyphenols and polysaccharides are as well responsible for their healing effects. Phenols and flavonoids are also antioxidative agents of *P.major* that contribute to wound healing by protecting the cells from destruction by inflammatory agents [11]. In animal studies, the *P. major* crude extract likely speeded up healing activity and regeneration of the epidermis layer [13, 14]. *P.major* also seems to have antiulcerative activities; however, the mechanism of action is not fully understood, but the healing effect of leaf extracts on gastric ulcers of rat models was significant, and it is possible that this compound contains anti *H.pylori* activity [11, 15]. Furthermore, this plant has demonstrated cytoprotective activities against viruses, which are likely due to chlorogenic acid and caffeic acid [16]. Reduction in the number of bacteria in mice serum and inhibition of bacterial growth by a disk diffusion method is other antimicrobial capacities that are suggested to be the effect of polysaccharide compounds [17]. Anticancer, antiurolithiasis, antinociception, renoprotective effects, antidiabetes, antidiarrhea, and antifatigue properties have been investigated in studies as well [11].

Considering that *P. major* plants are available all over the world and have several medical applications in topical and oral forms without considerable side effects, they could be utilized as a potential alternative therapy for burn injuries [10].

To the best of our knowledge, *P.major* topical application on human burn wounds has not yet been investigated in modern medicine [10]. So, we conducted research based on previous animal studies to evaluate the *P. major* effect on the duration of wound healing, pain control, and infection in patients diagnosed with partial-thickness burn wounds who were referred to Amir Al-Momenin Hospital at Shiraz, Iran.

## 2. Materials and Methods

### 2.1. Study Design and Participants

This case-control trial was conducted on burn patients admitted at Amir Al-Momenin Burn and Wound Healing Hospital for a period of nine months. The inclusion criteria consisted of burn patients with a second-degree burn between 18 and 60 years of age and a body mass index between 18 and 25 and who endured a burn injury with flame or hot liquid during the past 24 hours. The exclusion criteria consisted of patients with a dermatological disorder, pregnancy, a history of sensitivity to herbal medicine, addiction, use of medication interfering with the wound-healing process such as immunosuppressive or cytotoxic, or any comorbid disease, which could affect the wound-healing process, such as liver or renal failure, pulmonary or cardiovascular disorders, diabetes, anemia, severe malnutrition, cancer and malignancies, immunodeficiency, toxicity, trauma, intubation, rheumatological and vascular disorders, or vasculitis, or did not consent to participate in the study. Patients who developed severe drug reactions, visible pus discharge, or signs of active infection during the study were also excluded. Each patient was also compared with themselves as a control subject. We selected burn wounds that compromised 2 to 10% of the body while also selecting their symmetrical counterpart as the control.

Eligible individuals underwent initial examinations for sensitivity tests of *P.major* ointment. The aim of the study, along with the possible side effects and summarization of the findings of previous studies on this drug, was explained to each patient by the researcher, and subsequently, written informed consent was obtained; the patient's information was recorded in a preprepared datasheet.

### 2.2. Intervention

The dressing method was similar in both intervention and control groups, and the medication was administered through an unlabeled similar container to ensure that the patient and administrant were blinded to the grouping. The burn wound was first washed with sterile normal saline serum and using an abaisse-langue, a thin layer (5 gr/each percentage burn injury) of *P.major* ointment in the intervention group, and silver sulfadiazine ointment 1% in the control group was rubbed on sterile gauze, and the wound was bandaged with these gases. The patient's pain was recorded based on the visual analog scale (VAS) on the first, third, seventh, and tenth days of hospitalization, with the patient being asked to rate his pain on a scale of 1 to 10 (10 highest and 1 lowest pain). To study the process of infection control, in both groups on days 3, 7, 10, and finally 13 in case of no improvement, the culture was sent from the patient's wound.

The patient's dressing was changed daily until the wound was completely healed in such a manner that the wound has a pink appearance, clear, without discharge, and with granulation tissue and repaired epithelium. The dressing was changed by two trained and specific nurses who were blind to the research project and supervised by the head of the ward, a surgeon, and an infectious disease specialist daily. The examining physician did not know the type of dressing performed and ultimately concluded the diagnosis of complete wound healing. Variables such as the duration of healing, the pain intensity, and the incidence of wound infection were recorded and subsequently analyzed.

*P. major* plant ointment was prepared based on research on rats [18] and according to the opinion and prescription of the Department of Pharmacology of Shiraz School of Pharmacy. The plant was collected from Neyriz District, Fars Province, Southern Iran, by the collaboration of the school of pharmacy professors and local experts. The dried leaves and branches of the plant were ground into a fine mill and then mixed with a ratio of “1/20” in sterile water and subsequently placed in a hotplate for three hours, and after cooling, they were passed through a suitable filter. The extract was frozen and dried and placed in Vaseline with a concentration of 10%, and ultimately, a hydroalcoholic extract of *P.major* was prepared, which was evaluated in terms of efficiency and dry weight of the extract, while the sterility of the product was also investigated.

### 2.3. Statistical Analysis

The obtained data were statistically analyzed using SPSS software version 21. The Kolmogorov–Smirnov test was used to determine the normality of the distribution of all quantitative data and to compare the findings between the groups, and the independent *t*-test and repeated measures analysis of variance (ANOVA) were used. In this study, *P* < 0.05 was considered statistically significant. To remove statistical bias, the study was performed in a double-blind manner.

## 3. Results

**Figure 1:**
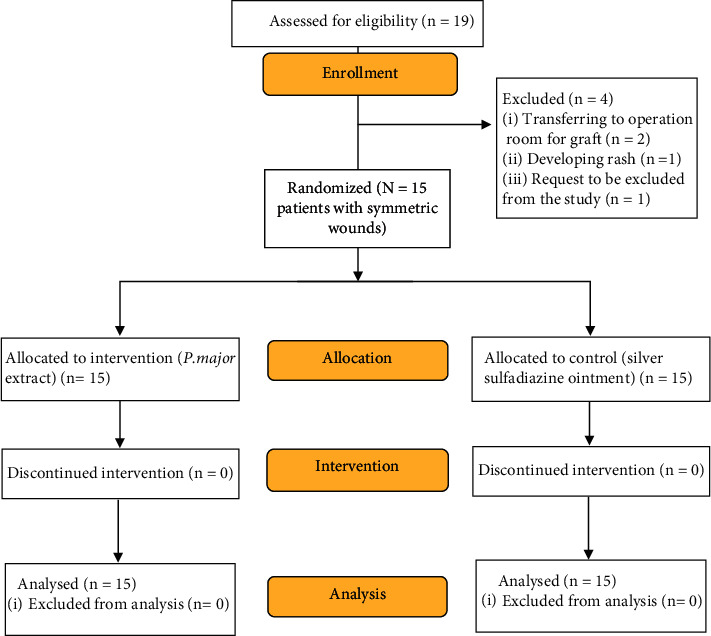
CONSORT flow diagram of burn patients receiving *P.major* extract or silver sulfadiazine ointment 1%.

Of 19 patients with burn injuries, four were excluded, leaving a total of 15 evaluated in our study (Figure 1). Among them, 11 were male (73.3%) and four were female (26.7%), and the mean age of the patients was 33.3 (range 21–49) years. Each patient fulfilled the inclusion criteria and had a symmetric burn injury, and one side was treated as the intervention group (wounds 1–15I) and the other side was treated as the control group (wounds 1–15°C). Each wound was subsequently compared and evaluated with its counterpart. Demographic characteristics and the baseline of patients are similar to the intervention and control groups since in this study, each patient was self-controlled.

**Figure 2:**
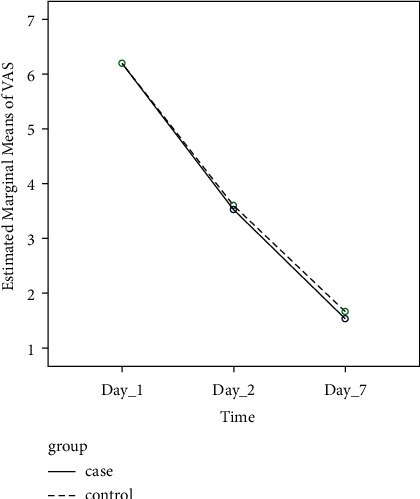
Line chart of the pattern of pain changes on different days between the intervention and control groups.

The mean recovery time in the intervention group was 11.73 ± 2.22 days, and in the control group, it was 13 ± 2.65 days. The results of this test showed that the mean recovery time between the two groups was not statistically significant and was similar (*P* value = 0.166), To compare the infection control between the two groups, considering that the wound culture on the seventh day onwards is all negative, we only compare the infection control on the third day between the two groups, which showed that on the third day, both groups were similar. Therefore, ten people had the positive infection culture (66.7%) and five people had the negative culture (33.3%), and in terms of infection control, the two groups are similar.  Table 1 demonstrates the recovery time, infection rates, and VAS scores among the patients in our study. Also, Figure 2 demonstrates the pattern of pain changes on different days between the intervention and control groups.

Regarding VAS pain scores, there was no significant difference among the two groups of intervention and control on day 1 (6.2 *vs.* 6.2), day 3 (3.53 *vs*. 3.60), and day 7 (1.53 *vs*.1.66). (*P* value = 0.849). When evaluating the changes in scores throughout the study, both groups demonstrated a significant decrease in the VAS scores; however, based on ANOVA with repeated measures, when comparing both groups, this amount of decrease in VAS scores throughout the study was not statistically different among the two groups (6.2 to 1.5 and 6.2 to 1.6 in the intervention and control group, respectively; *P* value <0.001 intragroup, *P* value = 0.956 intergroup).

**Figure 3:**
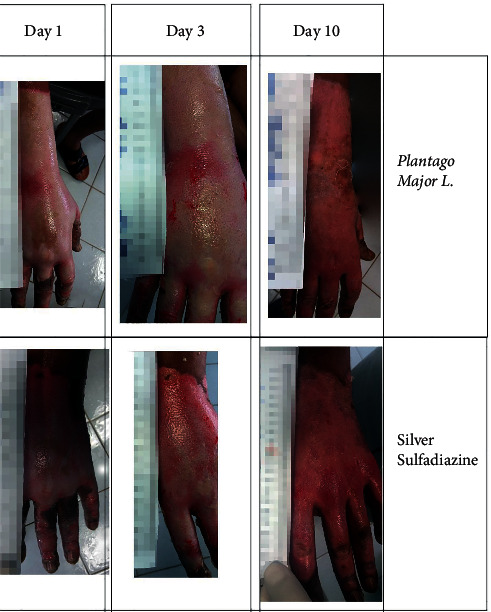
Comparison of the treatment results of burn injuries with Plantago major with silver sulfadiazine in a 28-year-old male with 9% burn injury due to gas capsule explosion.

**Figure 4:**
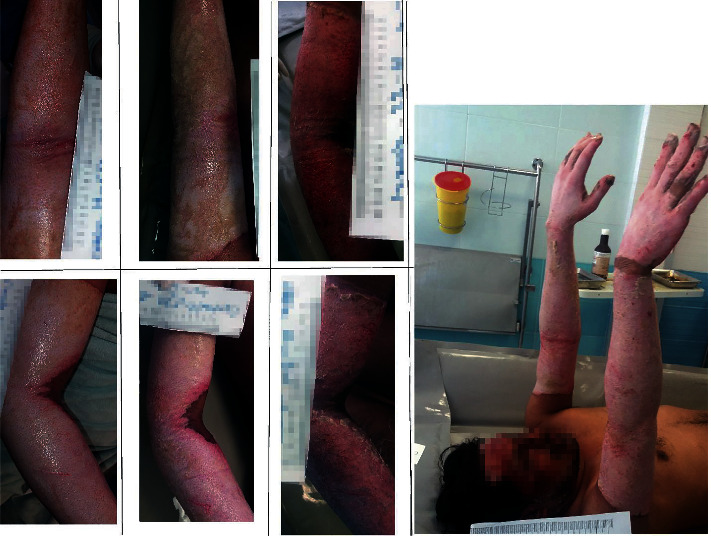
Evaluation of the treatment results of burn injuries with Plantago major (top row and left hand) in comparison to silver sulfadiazine (bottom row and right hand) in a 36-year-old male with 36% burn injury due to gas capsule explosion.

It is also worth mentioning that no noticeable side effects of dermatological conditions were observed in the patients following the treatment with *P.major*. Figures 3 and 4 demonstrate the treatment with *P.major* and silver sulfadiazine among two of the patients in our study.

## 4. Discussion

*P. major* plant has a long history in traditional medicine in the treatment of several diseases [10]. From the perspective of modern medicine, this plant contains a number of safe compounds.

With minimal adverse reactions and several medical benefits [10], it is believed that quality wound dressing is the main treatment in partial-thickness burn injuries and silver sulfadiazine is the first choice for topical therapy. Considering the adverse effects of sulfadiazine, several topical agents are suggested for wound dressing. [19] The current study supports the idea that the application of the *P. major* extract is effective in wound healing as compared to silver sulfadiazine.

In our study, the recovery time of *the P. major* administered group was shorter than that of the silver sulfadiazine group, although not significant. Despite the lack of human studies on this topic, there are animal research studies that support the healing properties of *P. major*. In this regard, an animal study by Amini et al. indicated good re-epithelialization, low inflammatory cell concentration, and well granulation tissue formation of burn wounds of rats treated with *P. major* [20]. They reported that the *P. major* water extract, especially with 50% concentration, could be a suitable substitute for sulfadiazine silver in burn injury healing and also suggested further studies in humans [20]. Another study demonstrated that the mixture of *P. major* and Aloe vera gel speeded up the wound recovery time as the result of an increase in the density of vessels, fibroblasts, and collagen fibers in the wound site [21]. Furthermore, in a human study, this gel was found to have significantly reduced the ulcer surface of diabetic foot ulcers [22]. An *in vitro* scratch study in 2012 investigated the effect of water and ethanol extracts of dry and fresh leaves of *P. major* on oral epithelial cells. Results showed that proliferation and migration of epithelial cells increased with all *P. major* extracts, leading to faster wound healing. An ethanol-based extract, which was also used in our study, demonstrated the strongest effect [23].

Our study proved the antiseptic property of *P. major* in wound healing. On the third day of our study, 66.7 cultures were positive, but infection of the case and control groups was similar, and from the seventh day, no wound infection was noted. Several authors have mentioned *P. major* as an antibacterial, antifungal, and antiviral compound. A study in Turkey demonstrated that the acetone extract of *P. major* has antimicrobial properties against nine species of bacteria, and also, an ethyl alcohol extract has bactericidal activity against *E. coli* and *Bacillus* cereus [24]. Fungicidal activity of *P. major* against growth, metabolic activity, and biofilm formation of Candida albicans was proved in a study by Shirley et al. [25]. The *P. major* extract also has antiviral activities; among a number of compounds of this plant, caffeic acid had the strongest antiviral effect against herpes virus and chlorogenic acid exhibited the strongest effect against adenovirus [26].

Analgesic and anti-inflammatory effects of *P. major* seem to be associated with inhibition of synthesis of prostaglandins [27]. In the current study, significant pain reduction was noted in both groups. A study in 2018 among cancer patients with oral mucositis showed that *P. major* was not only effective in the treatment of mucositis but also in reducing pain [28]. The same results were also seen among cancer patients with radiation-related oral mucositis who received this ingredient [29].

Regarding our study and the evaluation of *P. major*, some shortcomings should be addressed. First of all, what we know about the *P. major* mechanism of action in wound healing is limited to a few animal studies; however, few human studies have used *P. major* in the treatment of diabetic foot ulcer and oral mucositis [22, 28, 30]. We only investigated second-degree burn wounds, and the effect of this compound on the treatment of third and fourth-degree burn wounds is unclear. Also, the long-term effects of *P.major* on the wound site are not available because of lack of follow-ups after trials. Among the other, limitations of our study is the small number of evaluated patients, and the lack of performing a histopathological evaluation of the injury sites. To come to stronger conclusions, we recommend research with a larger sample size that compares the *P. major* effect not only with sulfadiazine but also with other antiseptic and topical wound healers.

## 5. Conclusion

Our study shows that *P. major* is effective in second-degree burn wound healing, while it is also accompanied by antiseptic and analgesic effects. Regarding that this ingredient is safe, cheap, and available all over the world, we should consider it as a possible alternative in topical therapy of burn injuries. *P. major* containing topical could be a safe and effective alternative for silver sulfadiazine in the healing of second-degree burn wounds.

## Figures and Tables

**Table 1:** Analysis of variance of repeated measures to compare the pattern of pain changes on different days between the intervention and control groups.

Variable		Group		*P* value^*∗*^
		Intervention, *n=15*	Control, *n=15*	
Recovery time (day), mean (standard deviation)		11.73 (2.22)	13 (2.65)	0.166

Infection 3^rd^ day, *n* (%)	Positive	10 (66.6%)	10 (66.6%)	1.000
	Negative	5 (33.3%)	5 (33.3%)	

Infection 10^th^ day, *n* (%)	Positive	0 (0%)	0 (0%)	1.000
	Negative	15 (100%)	15 (100%)	

Visual analog score, mean (standard error) (95% confidence interval)	Day 1	6.200 (0.380) (5.421–6.979)	6.200 (0.380) (5.421–6.979)	Time: <0.001 Group: 0.849 Time^*∗*^group: 0.956
	Day 3	3.533 (0.329) (2.860–4.206)	3.600 (0.329) (2.927–4.273)	
	Day 7	1.533 (0.162) (1.201–1.866)	1.667 (0.162) (1.334–1.999)	

The symbol ^*∗*^indicates the independent sample *t*-test, chi-square, and repeated analysis of the variance test.

## Data Availability

The datasets used and analyzed during the current study are available from the corresponding author on reasonable request.

## References

[1] Bailey M., Sagiraju H., Mashreky S., Alamgir H. (2019). Epidemiology and outcomes of burn injuries at a tertiary burn care center in Bangladesh. *Burns*.

[2] Saeidinia A., Keihanian F., Lashkari A. P. (2017). Partial-thickness burn wounds healing by topical treatment: a randomized controlled comparison between silver sulfadiazine and centiderm. *Medicine*.

[3] Latifi N.-A., Karimi H., Motevalian S. A., Momeni M. (2017). Economical burden of burn injuries in a developing country. *Journal of Burn Care and Research*.

[4] Mohammadi A. A., Hoghoughi M. A., Karoobi M. (2021). Socioeconomic features of burn injuries in southern Iran: a cross-sectional study. *Journal of Burn Care and Research*.

[5] Mohammadi A. A., Karoobi M., Erfani A. (2020). Suicide by self-immolation in southern Iran: an epidemiological study. *BMC Public Health*.

[6] Mohammadi A. A., Keshavarzi A., Erfani A., Modarresi M.-S., Shahriarirad R., Ranjbar K. (2020). Evaluation of epilepsy and burn patterns in a tertiary hospital in southwestern Iran. *Epilepsy and Behavior*.

[7] Jaladat A.-M., Ranjbar K., Shahriarirad R., Salehi Z. (2021). Successful use of “ma’oljobon”, a persian medicine product, in a patient with severe chronic cough: a case report. *Advances in Integrative Medicine*.

[8] Sarkari B., Mohseni M., Moein M. R., Shahriarirad R., Asgari Q. (2017). Effect of hydroalcoholic extract of *Echinacea purpurea* in combination with meglumine antimoniate on treatment of *Leishmania* major-induced cutaneous leishmaniasis in BALB/c mice. *International Journal of Applied and Basic Medical Research*.

[9] Sarkari B., Sattari H., Moein M. R., Tamadon A. M., Rad R. S., Asgari Q. (2016). Effect of topical gel prepared with hydroalcoholic extract of *Echinacea purpurea* on treatment of *Leishmania major*-induced cutaneous leishmaniasis in BALB/C mice. *Journal of Pharmaceutical Negative Results*.

[10] Najafian Y., Hamedi S. S., Kaboli Farshchi M., Feyzabadi Z. (2018). *Plantago major* in Traditional Persian Medicine and modern phytotherapy: a narrative review. *Electronic Physician*.

[11] Adom M. B., Taher M., Mutalabisin M. F. (2017). Chemical constituents and medical benefits of *Plantago major*. *Biomedicine and Pharmacotherapy*.

[12] Samuelsen A. B. (2000). The traditional uses, chemical constituents and biological activities of *Plantago major* L. A review. *Journal of Ethnopharmacology*.

[13] Zubair M., Nybom H., Lindholm C., Brandner J. M., Rumpunen K. (2016). Promotion of wound healing by *Plantago major* L. leaf extracts–ex-vivo experiments confirm experiences from traditional medicine. *Natural Product Research*.

[14] Thome R. G., Santos H. B. D., Santos F. V. D. (2012). Evaluation of healing wound and genotoxicity potentials from extracts hydroalcoholic of *Plantago major* and *Siparuna guianensis*. *Experimental Biology and Medicine*.

[15] Rahimi R., Shams-Ardekani M. R., Abdollahi M. (2010). A review of the efficacy of traditional Iranian medicine for inflammatory bowel disease. *World Journal of Gastroenterology*.

[16] Chiang L., Chiang W., Chang M., Ng L., Lin C. (2002). Antiviral activity of *Plantago major* extracts and related compounds *in vitro*. *Antiviral Research*.

[17] Hetland G., Samuelsen A. B., Loslash Vik M. (2000). Protective effect of *Plantago major* L. Pectin polysaccharide against systemic *Streptococcus pneumonae* infection in mice. *Scandinavian Journal of Immunology*.

[18] Mahmood A., Phipps M. (2006). Wound healing activities of *Plantago major* leaf extract in rats. *International Journal of Tropical Medicine*.

[19] Jeschke M. G., Van Baar M. E., Choudhry M. A., Chung K. K., Gibran N. S., Logsetty S. (2020). Burn injury. *Nature Reviews Disease Primers*.

[20] Amini M., Kherad M., Mehrabani D., Azarpira N., Panjehshahin M. R., Tanideh N. (2010). Effect of *Plantago major* on burn wound healing in rat. *Journal of Applied Animal Research*.

[21] Ashkani-Esfahani S., Khoshneviszadeh M., Noorafshan A. (2019). The healing effect of *Plantago major* and Aloe vera mixture in excisional full thickness skin wounds: stereological study. *World Journal of Plastic Surgery*.

[22] Najafian Y., Khorasani Z. M., Najafi M. N., Hamedi S. S., Mahjour M., Feyzabadi Z. (2019). Efficacy of aloe vera/*plantago major* gel in diabetic foot ulcer: a randomized double-blind clinical trial. *Current Drug Discovery Technologies*.

[23] Zubair M., Ekholm A., Nybom H., Renvert S., Widen C., Rumpunen K. (2012). Effects of *Plantago major* L. leaf extracts on oral epithelial cells in a scratch assay. *Journal of Ethnopharmacology*.

[24] Özkan O., Meti̇ner K., Ak S. (2012). Antibacterial effects of ethanol and acetone extract of *Plantago major* L. on gram positive and gram negative bacteria. *Kafkas Universitesi Veteriner Fakultesi Dergisi*.

[25] Shirley K. P., Windsor L. J., Eckert G. J., Gregory R. L. (2017). *In vitro* effects of *Plantago major* extract, aucubin, and baicalein on Candida albicans biofilm formation, metabolic activity, and cell surface hydrophobicity. *Journal of Prosthodontics*.

[26] Chiang L.-C., Chiang W., Chang M.-Y., Lin C.-C. (2003). *In vitro* cytotoxic, antiviral and immunomodulatory effects of *Plantago major* and *Plantago asiatica*. *The American Journal of Chinese Medicine*.

[27] Atta A., El-Sooud K. A. (2004). The antinociceptive effect of some Egyptian medicinal plant extracts. *Journal of Ethnopharmacology*.

[28] Cabrera-Jaime S., Martínez C., Ferro-García T. (2018). Efficacy of *Plantago major*, chlorhexidine 0.12% and sodium bicarbonate 5% solution in the treatment of oral mucositis in cancer patients with solid tumour: a feasibility randomised triple-blind phase III clinical trial. *European Journal of Oncology Nursing*.

[29] Soltani G. M., Hemati S., Sarvizadeh M., Kamalinejad M., Tafazoli V., Latifi S. A. (2020). Efficacy of the *Plantago major* L. syrup on radiation induced oral mucositis in head and neck cancer patients: a randomized, double blind, placebo-controlled clinical trial. *Complementary Therapies in Medicine*.

[30] Ghanadian M., Soltani R., Homayouni A., Khorvash F., Jouabadi S. M., Abdollahzadeh M. (2022). The effect of *Plantago major* hydroalcoholic extract on the healing of diabetic foot and pressure ulcers: a randomized open-label controlled clinical trial. *The International Journal of Lower Extremity Wounds*.

